# Assessing the role of spatial correlations during collective cell spreading

**DOI:** 10.1038/srep05713

**Published:** 2014-07-16

**Authors:** Katrina K. Treloar, Matthew J. Simpson, Benjamin J. Binder, D. L. Sean McElwain, Ruth E. Baker

**Affiliations:** 1 Mathematical Sciences, Queensland University of Technology (QUT), Brisbane, Australia; 2 Tissue Repair and Regeneration Program, Institute of Health and Biomedical Innovation, QUT, Brisbane, Australia; 3 Mathematical Sciences, University of Adelaide, Adelaide, Australia; 4 Wolfson Centre for Mathematical Biology, Mathematical Institute, University of Oxford, United Kingdom

## Abstract

Spreading cell fronts are essential features of development, repair and disease processes. Many mathematical models used to describe the motion of cell fronts, such as Fisher's equation, invoke a mean–field assumption which implies that there is no spatial structure, such as cell clustering, present. Here, we examine the presence of spatial structure using a combination of *in vitro* circular barrier assays, discrete random walk simulations and pair correlation functions. In particular, we analyse discrete simulation data using pair correlation functions to show that spatial structure can form in a spreading population of cells either through sufficiently strong cell–to–cell adhesion or sufficiently rapid cell proliferation. We analyse images from a circular barrier assay describing the spreading of a population of MM127 melanoma cells using the same pair correlation functions. Our results indicate that the spreading melanoma cell populations remain very close to spatially uniform, suggesting that the strength of cell–to–cell adhesion and the rate of cell proliferation are both sufficiently small so as not to induce any spatial patterning in the spreading populations.

Moving fronts of cells are frequently observed in embryonic development, tissue repair and cancer progression^1^,^2^,^3^,^4^,^5^,^6^. *In vitro* experiments, such as scratch or circular barrier assays, play an important role in identifying and quantifying the mechanisms that control the motion of such cell fronts^3^,^7^,^8^,^9^,^10^,^11^,^12^,^13^. Standard continuum models, such as Fisher's equation or generalisations thereof, are often used to describe the motion of *in vitro* cell fronts^3^,^5^,^9^,^10^,^13^,^14^,^15^. However, these models invoke a mean–field assumption implying that there is no underlying spatial structure, such as cell clustering, present in the system^16^,^17^.

It is well known that strong cell–to–cell adhesion or sufficiently rapid cell proliferation can lead an initially uniform population of cells to become clustered over time^17^,^18^. Our previous work has compared averaged discrete simulation data with predictions from standard mean–field descriptions of these discrete simulations for systems where either strong adhesion^19^ or rapid proliferation is present^20^,^21^. These previous comparisons have confirmed that standard mean–field models fail to accurately predict the averaged behaviour of the discrete model which implies that the usual mean–field assumption is inappropriate where either strong cell–to–cell adhesion or rapid proliferation is present^17^,^19^,^20^,^21^. We do not aim to repeat these kinds of comparisons between averaged discrete simulation data and the predictions of a mean–field model in this work. Instead, we analyze a detailed experimental data set with the aim of demonstrating how the presence of spatial structure, such as cell clustering, can be identified and quantified.

Unlike mean–field models, individual–based models explicitly incorporate spatial correlation effects^20^,^21^,^22^ and allow us to visualise the cell spreading process in a way that is directly comparable with experimental images^10^,^11^,^23^,^24^. However, individual–based models are computationally expensive and many realisations are required to obtain reliable statistics, meaning that it is often difficult to simulate realistic biological systems^22^. Mean–field models are more amenable to analytical exploration and hence can be advantageous over individual–based models provided that the mean–field assumption is an accurate representation of the relevant system^17^,^22^.

It is not always clear which modelling framework is appropriate for a given context without first testing the underlying model assumptions. For example, spreading populations of 3T3 fibroblast cells do not generally exhibit visible cell clustering, whereas populations of MDA MB 231 breast cancer cells appear to be highly clustered^10^,^17^. At first glance, it may appear reasonable to use a mean–field model to describe the spreading of a population of 3T3 cells and a discrete model to describe the spreading of a population of MDA MB 231 cells. However, recent work has indicated that the presence or absence of spatial correlations can be difficult to detect visually and so our use of a mean–field model for 3T3 cell population spreading may, in fact, be inappropriate^18^. Consequently, applying diagnostic tools which are capable of identifying spatial structure in a given cell population may provide insights into which modelling frameworks are suitable for exploring a particular system.

Several methods have been developed to assess the degree of spatial correlations in populations including measurements of the coordination number, Ripley's K function and Moran's I statistic^21^,^25^,^26^,^27^,^28^. A specific measure of spatial correlations is the pair–correlation function, *F*(*r*), which describes how the probability of finding two objects at a given distance, *r*, relates to the the probability of finding two objects, separated by the same distance, in a spatially uniform population^17^,^18^,^25^. Pair–correlation functions are a useful tool as they can be used to distinguish between spatial patterns, such as aggregation or segregation, at various length scales^18^,^25^,^29^. In particular, pair–correlation functions have been successfully used to distinguish differences between spatial patterns of benign and malignant cells^30^.

In this work, we quantify the extent to which the location of individual MM127 melanoma cells^31^,^32^,^33^ are spatially correlated during an *in vitro* cell spreading assay. We perform several *in vitro* experiments where cells are initially placed in a circular barrier and then the population spreads outwards after the barrier is lifted^10^,^11^. In particular, we consider a detailed experimental procedure where all experiments are repeated under two different conditions: first, where cells are treated to prevent proliferation, and second, where cell proliferation is permitted. This is important because MM127 melanoma cells are known to be motile, adhesive and proliferative^11^, and our experimental procedure allows us to examine the effects of proliferation separately from adhesion. This therefore allows us to determine whether spatial correlations are present, and, if so, whether the spatial correlations are associated with cell proliferation or cell–to–cell adhesion^10^,^11^.

To assess the degree of spatial correlations in our experimental cell populations, we calculate the pair–correlation function developed by Binder and Simpson^25^, which accounts for volume exclusion (crowding) and is relevant when considering biological cells which cannot occupy the same location in space. We also examine the conditions under which spatial structure can form in a spreading cell population using discrete simulations that mimic the spreading melanoma cell population. Using the pair–correlation function we confirm that the distribution of cells is initially spatially uniform. Finally, we use the pair–correlation function to determine whether any spatial correlations over short length scales emerge during the cell spreading process. All experiments are repeated for two different initial cell densities. Our results confirm that the degree of cell motility, cell proliferation and cell–to–cell adhesion in the spreading melanoma cell populations does not lead to significant spatial correlations.

## Results

### Visual inspection of spreading MM127 melanoma cell populations does not provide insights into possible spatial correlations

Circular barrier assays were conducted to examine the role of spatial correlations in a spreading population of MM127 melanoma cells over a period of *t* = 48 hours^11^. The exact nature of the experiments is described in the methods section. Briefly, cells were initially placed inside a circular barrier and the barrier was then lifted allowing the cell population to spread outwards. To distinguish whether cell proliferation has a significant effect on the presence of spatial correlations in the cell population, we performed experiments with Mitomycin–C pretreatment to suppress cell proliferation^34^ and then repeated the experiments without Mitomycin–C pretreatment.

**Figure 1:**
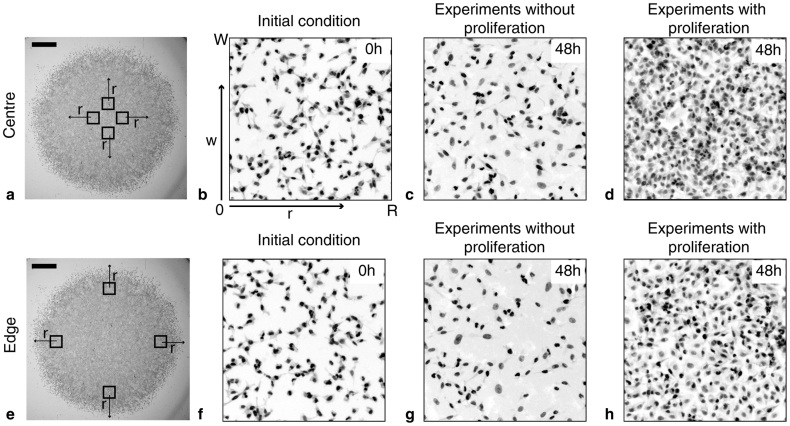
Experimental subregions of spreading MM127 melanoma cell populations. The role of spatial correlations in spreading MM127 cell populations was investigated by considering circular barrier assays initiated with 30,000 cells. For each experiment we calculated the pair correlation functions in four subregions, each of dimension 600 *μ*m × 600 *μ*m, at the centre of the spreading cell population and in four subregions, each of dimension 600 *μ*m × 600 *μ*m, at the edge of the spreading cell population. The relative size and approximate location of these subregions is shown in (a) and (e), where the scale bar corresponds to 1,500 *μ*m. Subregions showing the location of individual cells are shown at *t* = 0 hours in (b) and (f), at *t* = 48 hours for experiments without cell proliferation in (c) and (g), and at *t* = 48 hours for experiments with cell proliferation in (d) and (h). Note that the subregions in (b–d) and (f–h) are of dimension 300 *μ*m × 300 *μ*m. We describe the geometry of each subregion using coordinates (*r*, *w*), such that *r* indicates the direction of outward spreading and *w* measures the width of the subregion. The subregions in (a) and (e) correspond to 1 ≤ *r* ≤ 600 *μ*m and 1 ≤ *w* ≤ 600 *μ*m, while the regions in (b–d) and (f–h) correspond to 1 ≤ *r* ≤ 300 *μ*m and 1 ≤ *w* ≤ 300 *μ*m.

Figure 1 shows images of the entire spreading cell populations, as well as the relative location and size of various square subregions, each of dimension 600 *μ*m × 600 *μ*m, located both in the centre of the spreading population [Fig. 1 (a)] and towards the edge of the spreading population [Fig. 1 (e)]. Our analysis will focus on cell behaviour in these subregions. We also provide images, in Fig. 1, showing the distribution of individual cells within smaller subregions, of dimensions 300 *μ*m × 300 *μ*m, at the centre of the spreading cell population [Fig. 1 (b–d)] and at the edge of spreading cell population [Fig. 1 (f–h)]. For the purposes of analysis, *R* and *W* denotes the length and width of the subregion, respectively. Here, *r* corresponds to the radial distance in the direction of outward spreading (1 ≤ *r* ≤ *R*) and *w* corresponds to the direction perpendicular to *r* (1 ≤ *w* ≤ *W*). We expect an even distribution of individual cells at *t* = 0 hours since the experiments were initialised by placing cells as uniformly as possible inside the circular barrier^11^. Examining the snapshots at *t* = 0 hours, the cells appear to be spatially uniform with no visual evidence of clustering. However, without further analysis, it is difficult to conclude whether the cells are clustered or not^18^.

If we compare results at *t* = 48 hours in Fig. 1 (c–d) and (g–h), after cells have had the opportunity to migrate, adhere to other cells, and to proliferate, the cell populations still appear to be relatively uniform. However, it is difficult to conclude whether the cells are clustered or not simply from inspecting these snapshots. Comparing the snapshots where cell proliferation is permitted to those where cell proliferation is absent, it is clear that cell proliferation dramatically increases the density of cells but it is unclear whether there is any major change in the extent of cell clustering. Furthermore, comparing the snapshots of cells within the subregions located at the centre of the population with the subregions located towards the edge indicates that there is very little difference between the distributions of cells in these two different locations. Although there is no clear visual indication of spatial correlations, previous work^18^,^25^ suggests that further analysis should be undertaken before we can be certain that there is no underlying spatial structure present in the MM127 cell population.

### Discrete simulations of the experimental process provide insight into possible mechanisms inducing spatial correlations

Before we analyse the experimental images to quantify the role of spatial correlations, we first investigate how spatial correlations may emerge in the spreading MM127 melanoma cell populations by simulating the barrier assay using a discrete random walk model that incorporates cell motility, cell–to–cell adhesion and cell proliferation. We consider a two–dimensional model since the MM127 melanoma cell population spreads as a monolayer for the duration of the experiments^11^.

In this work, we considered two types of lattices; (i) a simulation lattice, and (ii) a pair correlation lattice. The simulation lattice, with lattice spacing Δ, is used to perform random walk simulations of the barrier assay. This involves modelling the spreading of a population of simulated cells, which mimic real cells in the experiments, undergoing motility events modulated by cell–to–cell adhesion, and proliferation events. Here, Δ is an indication of the average area that each individual cell occupies on the tissue culture plate. We chose to focus on the area occupied by the nucleus since the total area occupied by the cell fluctuates whereas the area occupied by the nucleus does not. To determine Δ, we measured the area of the nucleus and converted this into an estimate of the diameter of the nucleus (Δ ≈ 18 *μ*m, supplementary information).

The pair correlation lattice is used to compute the pair correlation function on a finer lattice, with lattice spacing *δ* = 1 *μ*m. Both experimental images and discrete simulation images are discretised onto the finer pair correlation lattice by resizing the dimensions of the image such that each pixel is 1 *μ*m × 1 *μ*m (supplementary information). Each pixel on the pair correlation lattice is either vacant (white pixel) or occupied (black pixel). Each black pixel is an object on the pair correlation lattice and corresponds to part of a cell in the experiments or part of a simulated cell in the discrete simulations. The advantage of discretising cells onto a pair correlation lattice using several black pixels (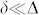) as opposed to discretising with one cell per lattice site is that we avoid having to select the location of individual cells on the lattice as this is not always an accurate representation of the original location of cells in the experiments^25^. The pair correlation signal is computed for all pair distances on the pair correlation lattice between 1 *μ*m and 600 *μ*m. For specific details of the calculation of the pair correlation function, *F*(*r*), we refer the reader to the methods section. When we present our estimates of the pair correlation function, *F*(*r*), we focus on pair distances in the interval 1Δ ≤ *r* ≤ 5Δ (18 *μ*m ≤ *r* ≤ 90 *μ*m) since we are primarily interested assessing spatial correlations over small to intermediate length scales^19^,^35^, but no smaller than the diameter of the nucleus^25^.

Random walk simulations are initialised to mimic the experimental procedure where either 20,000 or 30,000 cells are placed, uniformly at random, inside the circular barrier. Each circular barrier, of diameter 6,000 *μ*m, is placed into the centre of a well on a tissue culture plate. The well has a diameter of 15,600 *μ*m. To mimic this geometry in the discrete simulations we place either 20,000 or 30,000 simulated cells, uniformly at random, inside a circular region of diameter of 334 ≈ 6,000/18 lattice sites. This circular region is located approximately in the centre of a square lattice of side length 867 ≈ 15,600/18 lattice sites.

A random sequential update algorithm is used to perform the discrete simulations^36^. If there are *S*(*t*) simulated cells at time *t*, during the next time step of duration *τ*, *S*(*t*) simulated cells are selected at random, one at a time, and given the opportunity to move with probability *P_m_*(1 − *q*)*^a^*. Here, 0 ≤ *P_m_* ≤ 1 is the probability that an isolated simulated cell can move a distance Δ during the time interval *τ*, 0 ≤ *q* ≤ 1 is a measure of cell–to–cell adhesion strength, and *a* = 0, 1, 2, 3 or 4 is the number of occupied nearest–neighbour lattice sites of that simulated cell. If *q* = 0, there is no cell–to–cell adhesion and nearest neighbour simulated cells do not adhere to each other. As *q* increases, the strength of cell–to–cell adhesion increases, and the motion of nearest–neighbour simulated cells is reduced as the cells adhere more tightly to each other. A simulated cell at position (*i*Δ, *j*Δ) steps to (*i*Δ ± Δ, *j*Δ) or (*i*Δ, *j*Δ ± Δ) with each target site chosen with equal probability of 1/4. Since our model is an exclusion process, which explicitly incorporates crowding effects, any attempted motility event where the target site is occupied will be aborted. Once the *S*(*t*) potential motility events have been assessed, another *S*(*t*) simulated cells are selected at random, one at a time, and given the opportunity to proliferate with probability 0 ≤ *P_p_* ≤ 1. If the opportunity to proliferate is successful, the proliferative simulated cell attempts to deposit a daughter simulated cell at (*i*Δ ± Δ, *j*Δ) or (*i*Δ, *j*Δ ± Δ) with each target site chosen with equal probability of 1/4. Again, any attempted proliferation event where the target site is occupied will be aborted. We relate the parameters in the discrete model, *P_m_* and *P_p_*, to standard measures of the cell diffusivity, *D* = *P_m_*Δ^2^/(4*τ*), and the cell doubling time, *t_d_* = *τ* log*_e_*(2)/*P_p_*^11^. Our previous work, which did not include any measurement of spatial correlation, modelled the spread of MM127 melanoma cell population and indicated that we have *D* ≈ 248 *μ*m^2^/hour^11^.

**Figure 2:**
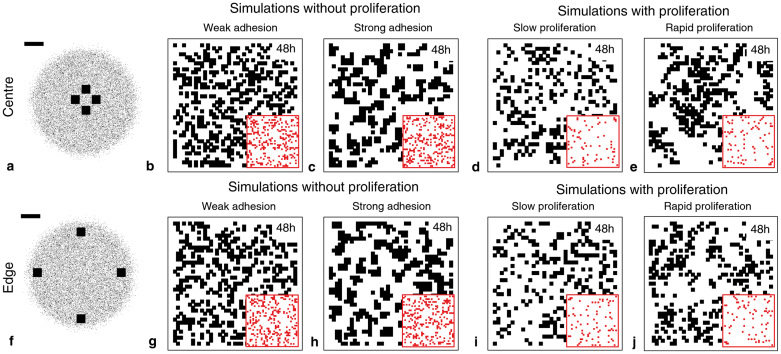
Discrete simulation snapshots with different combinations of cell motility, cell–to–cell adhesion and cell proliferation mechanisms. The emergence of spatial correlations in a spreading cell population was examined by simulating the biological process using a discrete random walk model with different combinations of adhesion, motility and proliferation. For each simulation we calculated the pair correlation functions in four subregions, each of dimension 600 μm × 600 μm, at the centre of the spreading cell population (a–e) and in four subregions, each of dimension 600 μm × 600 μm, at the edge of the spreading cell population (f–j). The relative size and approximate location of these subregions is shown in (a) and (f), where the scale bar corresponds to 1,500 μm. Simulations are performed on the simulation lattice where the lattice spacing, Δ = 18 μm, corresponds to the average diameter of the nucleus. Results in (b–c) and (g–h) correspond to simulations at *t* = 0 hours where 30% of simulation lattice sites are initially occupied with simulated cells, uniformly at random. While results (d–e) and (i–j) are initially occupied at 5%. The initial distribution of simulated cells, for each simulation, is shown as an inset in red. The size of the inset is approximately 550 μm × 550 μm. Simulation snapshots with no proliferation and weak adhesion (*q* = 0.3) are shown in (b) and (g) and snapshots with no proliferation and strong adhesion (q = 0.7) in (c) and (h). All results with no proliferation include unbiased motility where *D* = *P_m_*Δ^2^/4*τ* = 248 μm^2^/hour. Snapshots in (d) and (i) illustrate simulations with no adhesion and slow proliferation (*t_d_* = 23 hours). While results with no adhesion and rapid proliferation (*t_d_* = 12 hours) are shown in (e) and (j). Results with proliferation are simulated using *D* = 248 μm^2^/hour for *t_d_* = 23 hours and *D* = 23 μm^2^/hour for results with *t_d_* = 12 hours. Results in row 1 and 2 correspond to pair correlation signals computed at the centre and at the edge of the cell population, respectively.

**Figure 3:**
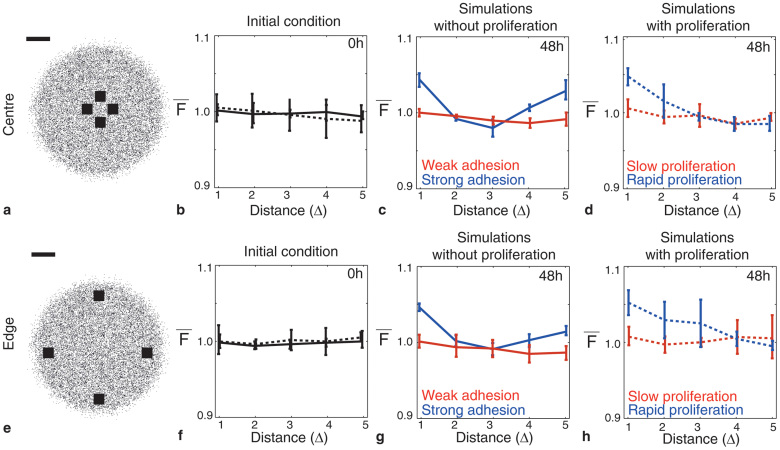
Different mechanisms in discrete simulations lead to varying pair correlation signals. Average pair correlation signals were computed from discrete simulations with varying degrees of cell–to–cell adhesion strength and cell proliferation in subregions, of dimension 600 *μ*m × 600 *μ*m, located at the centre and at the edge of the spreading simulated populations. The relative size and approximate location these subregions are shown in (a) and (e), respectively, where the scale bar corresponds to 1,500 *μ*m. Simulations are performed on the simulation lattice where the lattice spacing, Δ = 18 *μ*m, corresponds to the average diameter of the nucleus. Solid lines in (b–d) and (f–h) correspond to simulations without cell proliferation in which 30% of simulation lattice sites are initially occupied with simulated cells, uniformly at random. Dotted lines correspond to simulations with proliferation in which 5% of simulation lattice sites are initially occupied. Average pair correlation signals, constructed using *N* = 12 subregions from three replicate simulations, are shown at *t* = 0 hours in (b) and (f), at *t* = 48 hours for simulations without proliferation in (c) and (g), and at *t* = 48 hours for simulations with proliferation in (d) and (h). Pair correlation signals in (c) and (g) are shown for simulations with no proliferation and weak cell–to–cell adhesion (*q* = 0.3, red) and strong cell–to–cell adhesion (*q* = 0.7, blue). All results without proliferation include unbiased motility where *D* = *P_m_*Δ^2^/4*τ* = 248 *μ*m^2^/hour. Pair correlation signals for simulations with no adhesion and slow proliferation (*t_d_* = 23 hours, red) and rapid proliferation (*t_d_* = 6 hours, blue) are shown in (d) and (h). Results with proliferation are simulated using *D* = 248 *μ*m^2^/hour for *t_d_* = 23 hours and *D* = 23 *μ*m^2^/hour for results with *t_d_* = 12 hours. Results in row 1 and 2 correspond to pair correlation signals computed at the centre and at the edge of the cell population, respectively.

To understand how different mechanisms give rise to different spatial correlations in the discrete model, we simulated the spreading MM127 cell populations with varying degrees of cell motility (*D*), cell–to–cell adhesion strength (*q*) and cell proliferation (*t_d_*). Figure 2 shows several snapshots from the discrete model after *t* = 48 hours. In each snapshot, the initial distribution of simulated cells is shown as an inset. The corresponding average pair correlation functions, 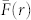, calculated using equation (9) (methods section), are shown in Fig. 3. In all cases, we analysed four subregions, of dimension 600 *μ*m × 600 *μ*m, both at the centre of cell population, as indicated by Fig. 3 (a), and four subregions at the edge of the cell population, as shown in Fig. 3 (e). Each spreading experiment was simulated using three identically–prepared realisations of the discrete model, giving a total of *N* = 3 × 4 = 12 identically prepared subregions. Pair correlation signals, 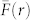, were computed from the discrete simulation data using exactly the same procedure that we apply to the experimental images, as described in the following section. The simulation lattice was resized onto the pair correlation lattice so that each lattice site corresponds to a physical length of *δ* = 1 *μ*m. This means that each square simulated cell is composed of 18 × 18 = 324 black pixels. Additional results indicate that the choice of *δ* is relatively insensitive provided that *δ* < Δ (supplementary information).

Results in Fig. 2 (b–c) and (g–h) mimic experiments with Mitomycin–C pretreatment in which cell proliferation is suppressed by setting *P_p_* = 0. Here, simulated cells undergo cell motility events modulated by cell–to–cell adhesion, but do not proliferate. Four subregions, each of dimension 600 *μ*m × 600 *μ*m, were considered at the centre of the cell population [Fig. 2 (a)] and at the edge of the cell population [Fig. 2 (f)]. The discrete snapshot at *t* = 0 hours, shown as an inset in Fig. 2 (b), appears spatially uniform, and this is confirmed by the corresponding pair correlation signal in Fig. 3 (b) which shows that 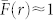 between 1Δ ≤ *r* ≤ 5Δ. If spatial correlations are present, we expect the pair correlation signal to deviate from unity^25^.

Discrete snapshots, after *t* = 48 hours, are shown in Fig. 2 for simulations with weak cell–to–cell adhesion [Fig. 2 (b) and (g)] and strong cell–to–cell adhesion [Fig. 2 (c) and (h)]. Visually we see that there is a significant difference in the spatial distribution of individual simulated cells when the strength of cell–to–cell adhesion is high. Here, simulated cells form clusters of around 5–15 individuals. In contrast, if we consider the case with weak cell–to–cell adhesion, the spatial distribution of individual simulated cells appears to be uniform and there are very few clusters. The corresponding pair correlation signals for each case, for subregions located at the centre of the cell population [Fig. 3 (c)], confirm our visual observations since 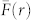 fluctuates around unity for simulations with weak cell–to–cell adhesion and deviates significantly from unity for simulations with strong cell–to–cell adhesion. The pair correlation signal for strong cell–to–cell adhesion indicates that 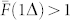 meaning that pairs of simulated cells at a distance of 1Δ are more probable than pairs of objects at the same distance in a spatially uniform population. The pair correlation signal at the edge of the population [Fig. 3 (g)] shows the same trend and illustrates that there is relatively little difference between the spatial distribution of cells at the centre and at the edge of the spreading population.

Similar results can be observed in Fig. 2 (d–e), (i–j) and Fig. 3 (d) and (h) where we show the results of simulations that mimic experiments without Mitomycin–C pretreatment and where cell–to–cell adhesion is not present (*q* = 0). Here, simulated cells undergo cell motility and cell proliferation events. In this case, we compare slow and rapid proliferation mechanisms where we observe that rapid cell proliferation leads to clustering. Here, 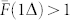 and 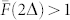, indicating that simulated cells at pair distances between 1Δ ≤ *r* ≤ 2Δ are more likely to occur than pairs of objects, separated by the same distance, in a spatially uniform population. To highlight the differences between slow and rapid proliferation, we obtained the results in Fig. 2 (d–e) and Fig. 2 (i–j) by initiating the simulations with a smaller number of simulated cells (5,000) than in the experiments. Furthermore, we also reduced the degree of motility in the simulations where we considered rapid proliferation. These differences were required otherwise the lattice becomes fully confluent after *t* = 48 hours with rapid proliferation and we note that a confluent monolayer of simulated cells has, by definition, no spatial structure. Therefore, reducing the initial number of cells and their motility rate allowed us to compare the spatial structure present at *t* = 48 hours before the lattice became confluent. The cell doubling time for MM127 melanoma cells is approximately 23 hours meaning that the total cell number will have approximately tripled over *t* = 48 hours in a modestly crowded environment. Hence, we expect in our experiments that the cell density, in regions away from the edge of cell population, will be approaching confluence by *t* = 48 hours. This means that any spatial correlations present in experiments with Mitomycin–C pretreatment could be masked by proliferation when it is not suppressed. This observation emphasises the importance of considering different experimental conditions to distinguish between the effects of different mechanisms^10^,^11^.

Our discrete simulation investigation indicates that cell populations where strong cell–to–cell adhesion or rapid cell proliferation are present are associated with spatial correlations and clustering which implies that the mean–field assumption is inappropriate to describe such systems^18^. The failure of the mean–field assumption to predict the averaged discrete behaviour for systems with either strong adhesion or rapid proliferation has been examined previously^19^,^20^,^21^. Although we know in advance that cell–to–cell adhesion and cell proliferation plays a role in governing the spreading of MM127 melanoma cell populations^11^, without any kind of analysis of the spatial distribution of individual cells within the population it is unclear whether these mechanisms are sufficiently strong to induce significant spatial correlations and clustering^11^.

### Spatial correlations are not present in spreading MM127 melanoma cell populations

Our experimental snapshots in Fig. 1 did not provide any conclusive visual evidence about whether spatial correlations may be present in the spreading melanoma cell populations. To quantitatively determine the extent to which the cell populations are spatially correlated, we computed the average pair correlation signals for all experiments using the same procedures applied to the discrete simulations, as discussed in the methods section. For each set of experiments, we analysed four subregions, each of dimension 600 *μ*m × 600 *μ*m, at the centre of cell population, as indicated by Fig. 1 (a), and four subregions, each of dimension 600 *μ*m × 600 *μ*m, near the edge of the cell population, as shown in Fig. 1 (e). Each experiment was repeated three times giving a total of *N* = 3 × 4 = 12 subregions. We note that each experimental subregion produces a similar pair correlation signal, *F*(*r*), over all pair distances considered in this work. Supplementary results illustrate that for each experiment and location considered, there are no obvious differences in the pair correlation signal across replicates or subregions. Hence, we treat each realisation as an identically prepared, independent subregion, and we determine the average pair correlation function, 
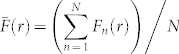
, where *N* = 12.

**Figure 4:**
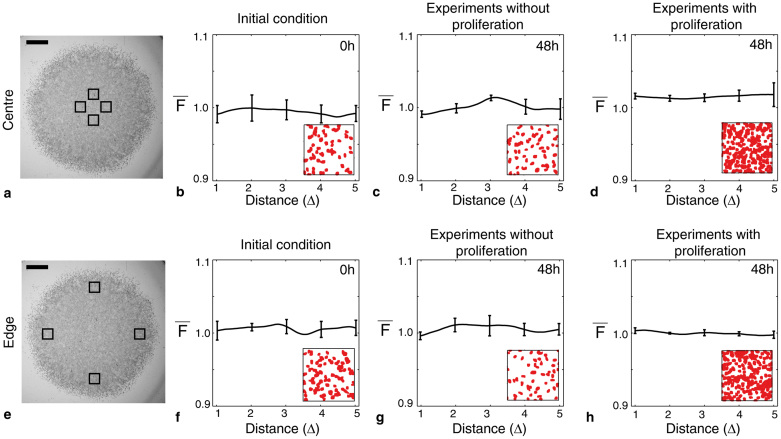
Spatial correlations are not present in spreading MM127 melanoma cell populations. Average pair correlation functions were extracted from images showing the location of individual cells in four subregions, each of dimension 600 *μ*m × 600 *μ*m, at the centre of the spreading cell population (a) and four subregions, each of dimension 600 *μ*m × 600 *μ*m, at the edge of the spreading cell population (e). The relative size and approximate location of these subregions is shown in (a) and (e), respectively, where the scale bar corresponds to 1,500 *μ*m. Average pair correlation signals are shown at *t* = 0 hours in (b) and (f), at *t* = 48 hours for experiments without cell proliferation in (c) and (g), and at *t* = 48 hours for experiments with cell proliferation in (d) and (h). Results in (b–d) and (f–h) correspond to pair correlation signals computed at the centre and at the edge of the spreading cell population, respectively. The horizontal axis is measured as multiples of the average diameter of the nucleus which is approximately 18 *μ*m. Snapshots of the experimental subregions after image processing are shown as an inset. The size of the inset is approximately 215 *μ*m × 215 *μ*m. Each pair correlation signal was averaged over 12 subregions of dimensions 600 *μ*m × 600 *μ*m, using three identically prepared experimental replicates. The error bars correspond to one standard deviation about the mean (*N* = 12). All experiments were conducted by initially placing approximately 30,000 cells inside the barrier assay.

Average pair correlation signals for all sets of experiments are shown in Fig. 4. Given that our experiments were initiated by placing cells as uniformly as possible inside the circular barrier at *t* = 0 hours, we expect that the pair correlation signal will fluctuate around unity (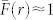) for all pair distances. The signals at *t* = 0 hours in Fig. 4 (b) and (f) confirm that the cells are initially distributed uniformly at random inside the barrier both at the centre of the cell population and at the edge of the cell population. Results after *t* = 48 hours, for subregions located at the centre [Fig. 4 (c–d)] and at the edge [Fig. 4 (g–h)] of the cell population, for all experiments with and without cell proliferation, also indicate that the average pair correlation signal, 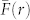, fluctuates around unity for pair distances between 1Δ ≤ *r* ≤ 5Δ.

The pair correlation signals in this work were computed using data extracted from experiments where 30,000 cells were placed inside the circular barrier initially. To investigate whether the initial cell density affects the presence of spatial correlations, we repeated the procedure using a different initial cell density where 20,000 cells were placed as uniformly as possible in the barrier and we found similar results (supplementary information). In addition to considering the pair correlation at the centre of the population and at the edge of the population, we also calculated the pair correlation signal at other locations across the spreading cell population. These additional results show that the pair correlation signal does not change significantly across the spreading cell population (supplementary information).

All results presented so far involve computing the pair correlation function, *F*(*r*), by considering distances between pairs of pixels in the direction of outward spreading, *r*. Alternatively, we could consider distances between pairs of pixels in the direction perpendicular to outward spreading, *w*, to give *F*(*w*). Additional results (supplementary information) compare *F*(*r*) and *F*(*w*), showing that the average pair correlation function is independent of the direction considered.

## Discussion

In this work, we investigated the presence of spatial correlations in a spreading population of MM127 melanoma cells by computing pair correlation signals at the centre and edge of the spreading cell population. Our results indicate that there is very little underlying spatial structure present in the experimental system. Assessing the presence of spatial correlations using statistical tools, such as the pair correlation function, allows us to quantify the degree to which spatial structure is present in a given cell population. This information may provide insight into which potential modelling frameworks could be used to represent the experimental system. The relative absence of spatial structure in the spreading MM127 melanoma cell populations implies that a mean–field model could be appropriate to represent these experiments, at least over the time scales explored in the experimental data set^16^,^17^.

Using our experimental data set, we have been able to investigate the relative roles of cell proliferation and cell–to–cell adhesion in terms of how they contribute to the formation of clustering. This is important because many experimental and modelling approaches neglect to consider the roles of adhesion and proliferation separately, meaning that it could be difficult to distinguish between the contributions of each mechanism^10^,^11^. We are interested in identifying the potential contribution of each mechanism since the analysis of the resulting spatial patterns from our discrete model indicates that both rapid proliferation and strong cell–to–cell adhesion can lead to significant spatial patterning and clustering. In contrast, our experimental results indicate that there were no major differences between the spatial distribution of cells in a population where cell proliferation was suppressed compared to the spatial distribution in a population where cell proliferation was present.

## Methods

### Cell culture

Human malignant melanoma cells (MM127,^31^,^32^,^33^) were cultured with 10% fetal calf serum (FCS), RPMI–1640, 2 mM L-Glutamine, 23 mM HEPES (Invitrogen, Australia) and 1% v/v penicillin/streptomycin (Invitrogen, Australia). Prior to confluence, cells were lifted using 0.05% trypsin–EDTA(1×) (Invitrogen, Australia) and viable cells were counted using a Trypan blue exclusion test and a haemocytometer.

### Circular barrier assay

The experimental procedure has been reported in detail previously^10^,^11^. Metal–silicone barriers (Aix Scientifics, Germany) were cleaned, sterilised, dried and placed in the centre of each well of a 24–well tissue culture plate. Experiments were performed using two different cell densities: 20,000 and 30,000 cells per well. Cell proliferation was suppressed in half of all cell solutions by adding 10 *μ*g/mL Mitomycin–C (Sigma Aldrich, Australia) for one hour at 37°C prior to transfer to the wells^34^. 100 *μ*L of cell suspension was carefully inserted into the barrier to ensure that the cells were approximately evenly distributed. Cells were allowed to settle and attach for four hours in a humidified incubator at 37°C, 5% CO_2_ and 95% air. Assays commenced with the removal of the barrier and the cell layer was washed with warm serum free medium (culture medium without FCS) and replaced with 0.5 mL of culture medium. Cultures were incubated at 37°C in 5% CO_2_ and 95% air for *t* = 0 and 48 hours. Each assay, for each time point, was repeated three times.

### Image acquisition and analysis

The cell nuclei were stained using 1 mg/ml Propidium Iodide (Invitrogen, Australia) in phosphate buffered saline and images were acquired using a Nikon Eclipse Ti inverted microscope fitted with a Nikon digital camera. Overlapping adjacent images were used to reconstruct a transect images detailing the location and size of individual cell nuclei along the spreading cell population. MATLAB's Image Processing Toolbox^37^ was used to convert the images into black and white by thresholding the image (*rgb2gray, imadjust, im2bw*). Images were discretised onto the pair correlation lattice by rescaling the image so that each square pixel corresponds to a length of *δ* = 1 *μ*m (*imresize*). White pixels correspond to unoccupied lattice sites and black pixels indicate occupied lattice sites. Each cell on the pair correlation lattice is composed of several black pixels. In all cases, a visual check was performed to validate that all cells had been correctly identified using the software. For discrete simulations, the simulation lattice was rediscretised onto the pair correlation lattice by scaling the lattice by a factor of 18 such that a simulated cell occupying one lattice site on the simulation lattice instead occupied 18 × 18 = 324 lattice sites on the pair correlation lattice and is composed of 324 black pixels.

### Pair–correlation function

Pair correlation functions were computed by considering pair distances between all black pixels on the pair correlation lattice for both experimental images and discrete simulation data^25^. The pair correlation lattice is a finite square lattice with integer coordinates, each site corresponding to the centre of a pixel and assigned coordinates (*r*, *w*), where 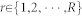 is a coordinate on an axis aligned in the direction of outward spreading and *w* ∈ {1, 2, …*W*} in the direction perpendicular to the direction of outward spreading. In our calculations we used *R* = *W*. The occupancy of black pixels on the pair correlation lattice is captured by the indicator function, 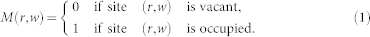
The number of black pixels (*n*) at any given time and the corresponding pair correlation density (*ρ*) are given by 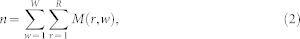

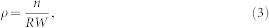


where *a* and *b* denote generic pixels with coordinates (*r_a_*, *w_a_*) and (*r_b_*, *w_b_*), respectively. We define the set of paired black pixels as 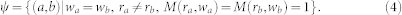
The subset of black pixel pairs at distance *i* (1 ≤ *i* ≤ *R*) is 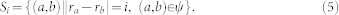
The number of elements in the subset *S_i_* indicate the counts of pair distances 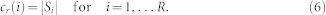
The normalisation factor is given by 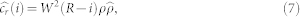
where 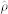 corresponds to the conditional probability of selecting the second black pixel in the black pixel pair given that the probability of selecting the first black pixel is the usual density *ρ*, 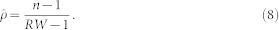
The pair–correlation function, *F*(*i*), is given by 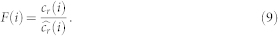
The pair–correlation function is calculated using *N* subregions giving an average pair–correlation function 
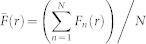
. If 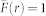, the probability of finding two black pixels at a given distance, *r*, is equal to the probability of finding two black pixels at the same distance in a spatially uniform distribution of objects^18^,^25^. If 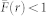, the probability of finding two black pixels at a given distance, *r*, is less than the probability of finding two black pixels at the same distance in a spatially uniform distribution of objects^18^,^25^. Alternatively, if 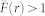, the probability of finding two black pixels at a given distance, *r*, is greater than the probability of finding two black pixels at the same distance in a spatially uniform distribution of objects^18^,^25^.

## Author Contributions

K.K.T. and M.J.S. conceived the study and designed the experiments. K.K.T. analysed the data. K.K.T. and M.J.S. wrote the manuscript. K.K.T., M.J.S., B.J.B., D.L.S.M. and R.E.B. revised the manuscript. All authors read and approved the final manuscript.

## Supplementary Material

Supplementary Information Supplementary information
