# Supplementary material for: Assessing the role of spatial correlations during collective cell spreading
Source: Sci Rep. 2014 Jul 16;4:5713. doi: 10.1038/srep05713 (PMC4100022; doi:10.1038/srep05713)
Supplement: Supplementary Information [file srep05713-s1.pdf]

SUPPLEMENTARY INFORMATION FOR 'ASSESSING THE ROLE OF SPATIAL CORRELATIONS DURING COLLECTIVE CELL SPREADING'

KATRINA K. TRELOAR*[1,2], MATTHEW J. SIMPSON[1,2], BENJAMIN J. BINDER[3], D.L SEAN MCELWAIN[2], AND RUTH E. BAKER[4]

S1. Estimating the total area occupied by individual MM127 melanoma cells 2
S2. Computing average pair correlation functions 3
S3. Average pair correlation signals for experiments with different initial cell densities 3
S4. Pair correlation signals in subregions located across the spreading cell population 6
S5. Insensitivity of pair correlation signal to δ 6
S6. Average pair correlation function in the w direction 6

* Corresponding author: Katrina K. Treloar (katrina.treloar@connect.qut.edu.au).
[1]Mathematical Sciences, Queensland University of Technology (QUT), Brisbane, Australia.
[2]Tissue Repair and Regeneration Program, Institute of Health and Biomedical Innovation, QUT, Brisbane, Australia.
[3]Mathematical Sciences, University of Adelaide, Adelaide, Australia.
[4]Wolfson Centre for Mathematical Biology, Mathematical Institute, University of Oxford, United Kingdom.

S1. Estimating the total area occupied by individual MM127 melanoma cells

In our work, we require an estimate of Δ, which approximates the diameter of the area occupied by a cell. During our experiments we observe that the shape of the cells constantly fluctuate with time. To account for this variation, we estimate the diameter of the cell nucleus since the fluctuations in the size of the nucleus appear to be much smaller than the fluctuations in the size of the entire cell and therefore provides us with a more reliable estimate of the average area occupied by each cell. Images of the cell nuclei were acquired using a Nikon TI Eclipse microscope fitted with a Nikon digital camera. Images were thresholded using MATLAB's image processing toolbox and discretised on a lattice by resizing the dimensions such that each pixel is 1 μm $\times$ 1 μm. Each pixel on the lattice is either vacant (white pixel) or occupied (black pixel) and a cell is composed of several black pixels. The process used is the same process used to discretise experimental images onto the pair correlation lattice in the main text.

Estimates of Δ were obtained by counting the number of black pixels per cell and converting this measurement into an area, A. We assume that, on average, the morphology of each cell is circular and we convert A into a diameter estimate using $2\sqrt{A/\pi}$. Fig. S1 (a) shows an image of MM127 cell nuclei and the corresponding discretisation onto a lattice (b). Table S1 summarises measurements for $n = 40$ cells and indicates that, on average, $\Delta \approx 18$ μm.

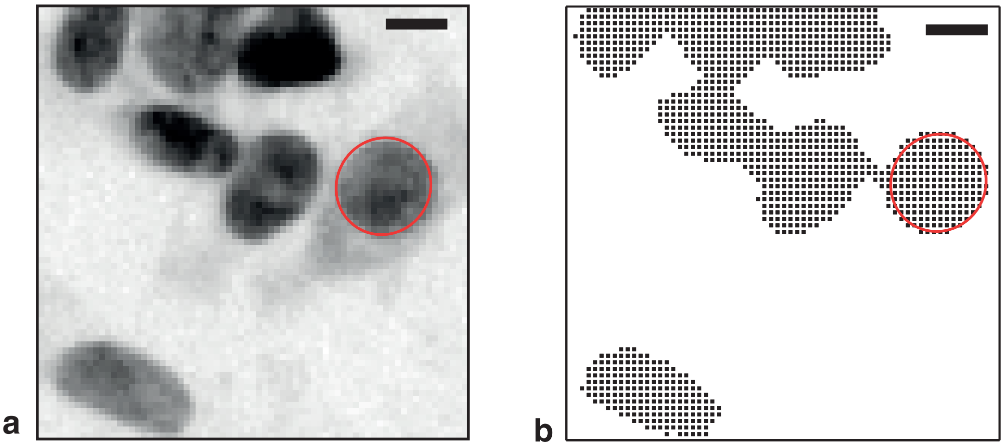

Figure S1. **Cell diameter measurements**. High magnification images of MM127 cells are shown in (a). Images were acquired using a Nikon Ti Eclipse microscope fitted with a Nikon digital camera. The scale bar corresponds to 10 μm. The nucleus diameter (b) of cells was determined by counting the number of black pixels of each cell in a discretised image, where each pixel corresponds to 1 μm $\times$ 1 μm and converting this into a circular measurement.

Diameter of cell nucleus (μm)	18.94 18.78 21.98 17.34 23.50 17.00 16.00 16.74 16.42 15.03<br>16.49 18.95 17.00 21.52 16.15 16.86 16.11 14.17 19.32 21.15<br>16.80 17.87 16.58 16.50 17.75 16.93 16.65 16.80 21.51 18.05<br>17.18 15.38 23.07 15.17 15.97 21.41 22.48 17.58 16.50 18.00
Mean (μm)	17.94
Standard deviation (μm)	2.37

Table S1. Cell nucleus diameter measurements of 40 MM127 cells indicate that the mean nucleus diameter of the cell, Δ, is 17.94 ± 2.37 μm

S2. Computing average pair correlation functions

In the main text, we present average pair correlation functions, $\overline{F}(r)$, for all experimental results. Here, we demonstrate that there are no obvious differences in the pair correlation signal between each subregion in an individual experiment. Furthermore, we also demonstrate that the averaging approach taken in the main manuscript is reasonable. Figure S2 presents results for subregions located at the centre of the cell population for experiments without cell proliferation and with cell proliferation. Pair correlation signals shown in Fig. S2 (b) and (f) illustrate the signal extracted from four subregions, of dimension 600 μm $\times$ 600 μm, using one experimental replicate. The approximate locations of these subregions are illustrated in Fig. S2 (a) and (e), respectively. We observe that each signal fluctuates around unity for all pair distances between $1\Delta \leq r \leq 5\Delta$. We note that there is some variability between the pair correlation signals. However, there does not seem to be obvious differences or trends in the data. Results in Fig. S2 (c) and (g) illustrate the average pair correlation signal, $\overline{F}(r)$, from three experimental replicates of the same experiment using four subregions in each replicate. Again the pair correlation signals fluctuate around unity for all pair distances without any obvious trends in the data. In addition our results illustrate that the pair correlation signal for each different replicate of the same experiment is similar. The final average pair correlation signals, averaged using 12 subregions from three experimental replicates of the same experiment, is shown in Fig. S2 (d) and (h). Our results confirm that there are no obvious differences in the pair correlation signal across experimental subregions or replicates indicating that our averaging approach used in the main manuscript is appropriate.

S3. Average pair correlation signals for experiments with different initial cell densities

The main text presents average pair correlation signals from all sets of experiments in which $30,000$ cells were initially placed inside the circular barrier. To investigate whether the initial cell density affects the presence of spatial correlations in the spreading cell populations, we repeated the procedure using a different initial cell density where $20,000$ cells were placed as uniformly as possible in the barrier and we found similar results. Results in Fig. S3 at $t = 0$ hours and after $t = 48$ hours, for subregions located at the centre and at the edge of the cell population, for all experiments with and without cell proliferation, indicate that the average pair correlation signal, $\overline{F}(r)$, fluctuates around unity for pair distances between $1\Delta \leq r \leq 5\Delta$. These results suggest that there is very little spatial structure and clustering present in the spreading MM127 melanoma cell populations.

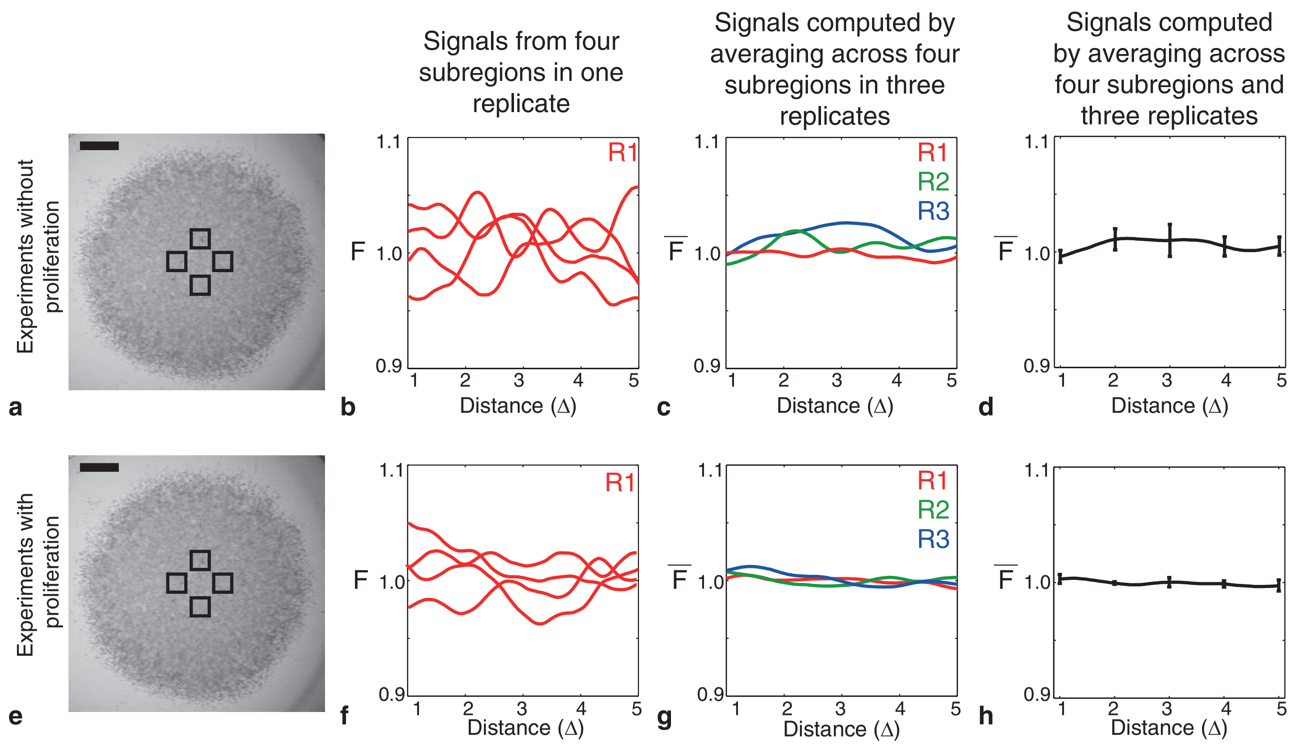

Figure S2. **Average pair correlation functions**. The role of spatial correlations in spreading MM127 cell populations was investigated by calculating pair correlation functions in four subregions, each of dimension 600 $\mu \times 600$ μm, at the centre of the spreading cell population and at the edge of the spreading cell population (e–h). The relative size and approximate location of these subregions is shown in (a) and (e), where the scale bar corresponds to 1,500 μm. Pair correlation functions were computed for experiments with Mitomycin–C pretreatment to suppress cell proliferation (b–d) and without Mitomycin–C pretreatment (f–h). Pair correlation signals were computed from four subregions of dimensions 600 μm $\times$ 600 μm and each individual realisation from replicate 1, $R1$, is shown in (b) and (f). The horizontal axis is measured as multiples of the average diameter of the nucleus. Averaging the pair correlation signals across four subregions for three experimental replicates is shown in (c) and (g), where, R1, R2 and R3 correspond to replicates 1, 2 and 3, respectively. Results in (d) and (h) illustrate the final pair correlation signal which is averaged across all 12 subregions from the three experimental replicates. The error bars correspond to one standard deviation about the mean ($N = 12$). All experiments were conducted by initially placing approximately 30,000 cells inside the barrier assay.

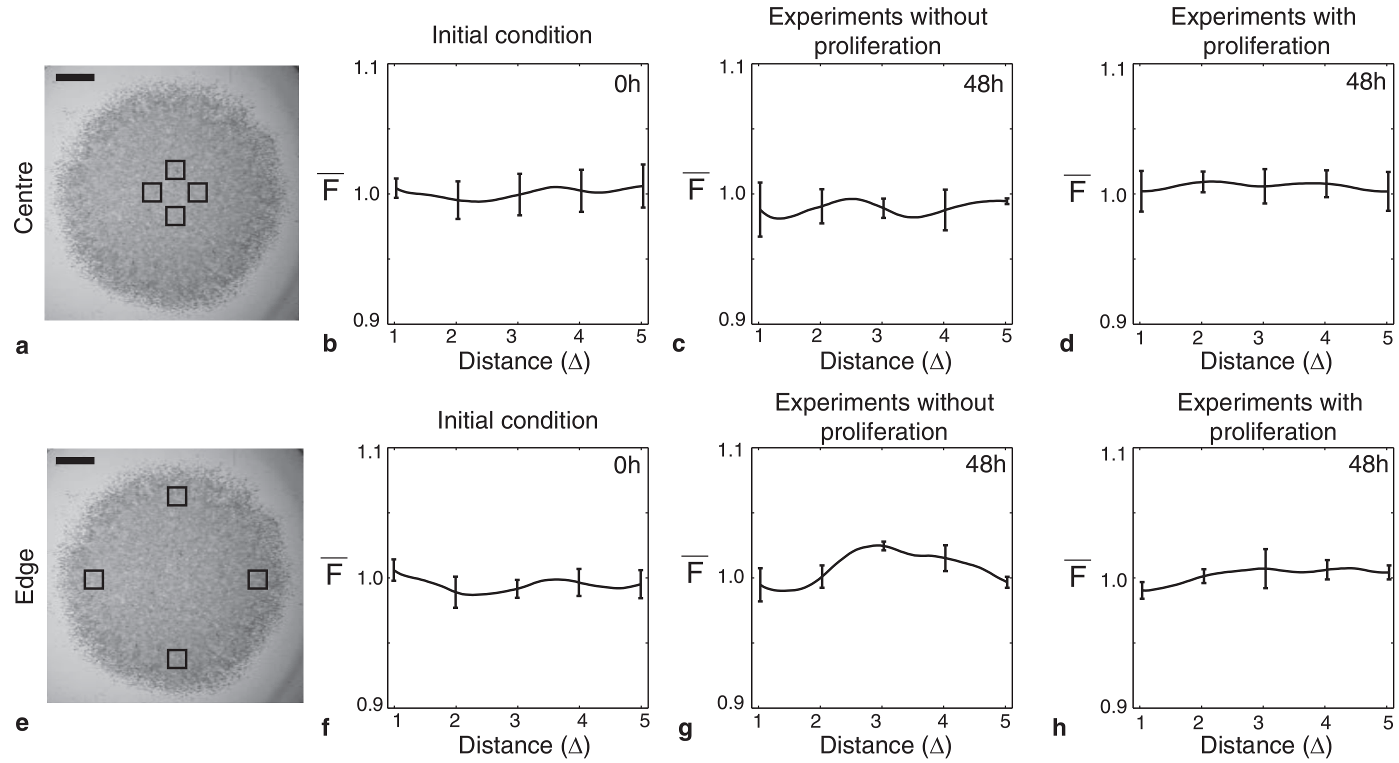

FIGURE S3. **Spatial correlations are not present in spreading MM127 melanoma cell populations**. Average pair correlation functions were extracted from images showing the location of individual cells in four subregions, each of dimension 600 μm $\times$ 600 μm, at the centre of the spreading cell population (a) and four subregions at the edge of the spreading cell population (e). The relative size and approximate location of these subregions is shown in (a) and (e), respectively, where the scale bar corresponds to 1,500 μm. Average pair correlation signals are shown at $t = 0$ hours in (b) and (f), at $t = 48$ hours for experiments without cell proliferation in (c) and (g), and at $t = 48$ hours for experiments with cell proliferation in (d) and (h). Results in (a–d) and (e–h) correspond to pair correlation signals computed at the centre and at the edge of the spreading cell population, respectively. The horizontal axis is measured as multiples of the average diameter of the nucleus. Each pair correlation signal was averaged over 12 subregions of dimensions 600 μm $\times$ 600 μm, using three identically prepared experimental replicates. The error bars correspond to one standard deviation about the mean ($N = 12$). All experiments were conducted by initially placing approximately 20,000 cells inside the barrier assay.

S4. Pair correlation signals in subregions located across the spreading cell population

We compute the pair correlation signal, in the main text, in subregions located at the centre and at the edge of the spreading cell populations. Our results show that there are no obvious differences in the pair correlation signal at these locations. To confirm that the pair correlation signal does not change significantly depending on the location of the subregion, we computed the pair correlation signal in five different subregions, each of dimension 800 μm $\times$ 800 μm, equally spaced between the centre and the edge of the cell population. The relative size and location of each of these subregions is illustrated in Fig. S4 (a–e). The corresponding pair correlation signal, using one experimental replicate, is shown in Fig. S4 (g–k), where we observe that the pair correlation signals, $F(r)$, fluctuates around unity for pair distances between $1\Delta \leq r \leq 5\Delta$ in each subregion. Our results illustrate that the pair correlation signals in subregions located across the spreading cell population are similar and we do not observe any obvious differences that depend on the location of the subregion. Hence, it seems reasonable that the pair correlation signals at each of these five subregions could be averaged to determine $\overline{F}(r)$. Results shown in Fig. S4 (f) and (l) illustrate the average pair correlation signal determined from the five subregions. The average pair correlation signal illustrates that $\overline{F}(r)$, fluctuates around unity for pair distances between $1\Delta \leq r \leq 5\Delta$ and that the standard deviation, shown by the error bars, is small confirming that the pair correlation signals extracted in subregions located across the spreading cell population are similar.

S5. Insensitivity of pair correlation signal to δ

In the main manuscript, we discretise experimental and discrete simulation images onto a finer pair correlation lattice by resizing the dimensions of the image such that each pixel is 1 μm $\times$ 1 μm. Here, the lattice spacing is $\delta = 1$ μm. To test whether the pair correlation signal is sensitive to δ, we repeated the process by discretising the images onto the pair correlation lattice using various values of δ between 0.1 μm $\leq \delta \leq$ 3 μm for the experimental images and 0.1 μm $\leq \delta \leq$ 18 μm for the discrete simulation images. For the experimental images, we do not consider any values of $\delta >$ 3 μm since we wish to avoid specifying or disrupting the physical location of the cells on the lattice. Results in Fig. S5 show two examples of the experimental and discrete images, shown inset in each subfigure, where $\overline{F}(r)$ has been computed for various values of δ. We observe that the δ values examined produce similar pair correlation signals.

S6. Average pair correlation function in the w direction

In the main manuscript spatial correlations in the spreading MM127 melanoma cell populations were assessed by considering distances between pairs of pixels in the direction of outward spreading, r, to give an estimate of the pair correlation signal, $F(r)$. For completeness, we now consider whether the pair correlation signals are sensitive to direction by repeating the analysis by considering distances between pairs of pixels in the direction perpendicular to the direction of outward spreading, w, to give an estimate of the pair correlation signal, $F(w)$. Results in Fig. S6 compare the corresponding average pair correlations signals, $\overline{F}(r)$ and $\overline{F}(w)$, confirming that the pair correlation signals in the intervals $1\Delta \leq r \leq 5\Delta$ and $1\Delta \leq w \leq 5\Delta$ are similar regardless of whether we analyse the r or w direction.

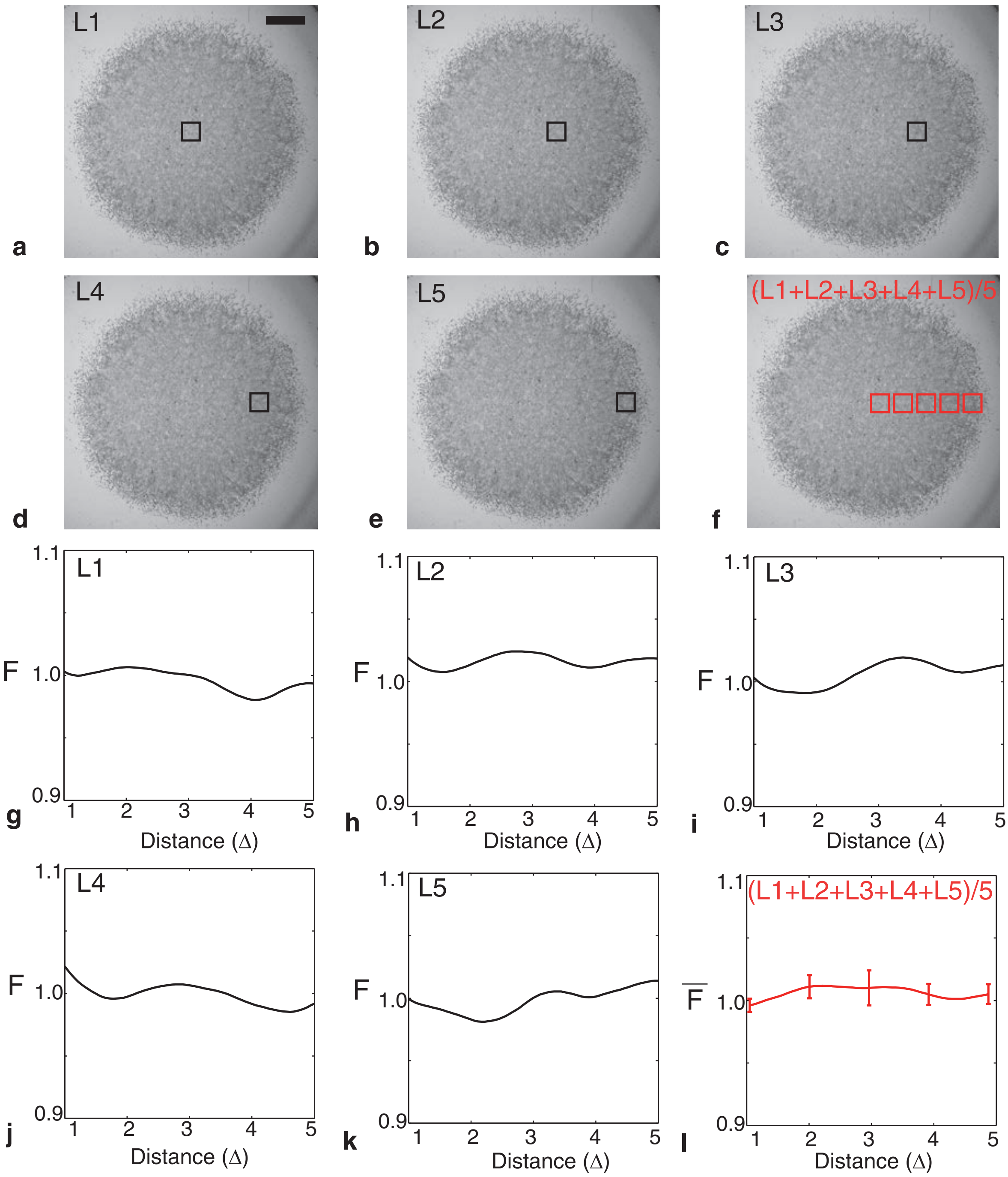

FIGURE S4. **Pair correlation signals are similar across the entire spreading cell population**. Pair correlation functions were extracted from images showing the location of individual cells in five subregions of dimensions, 800 μm $\times$ 800 μm located across the spreading cell population. The location of the subregion considered is shown in (a–e), where the scale bar corresponds to 1,500 μm. The corresponding pair correlation signal for each location is shown in (g–k). The horizontal axis is measured as multiples of the average diameter of the nucleus. The average pair correlation signal, calculated using $N = 5$ subregions across the spreading cell population as illustrated in (f), is shown in (l). The error bars correspond to one standard deviation about the mean ($N = 12$). All experiments were conducted by initially placing approximately 30,000 cells inside the barrier assay.

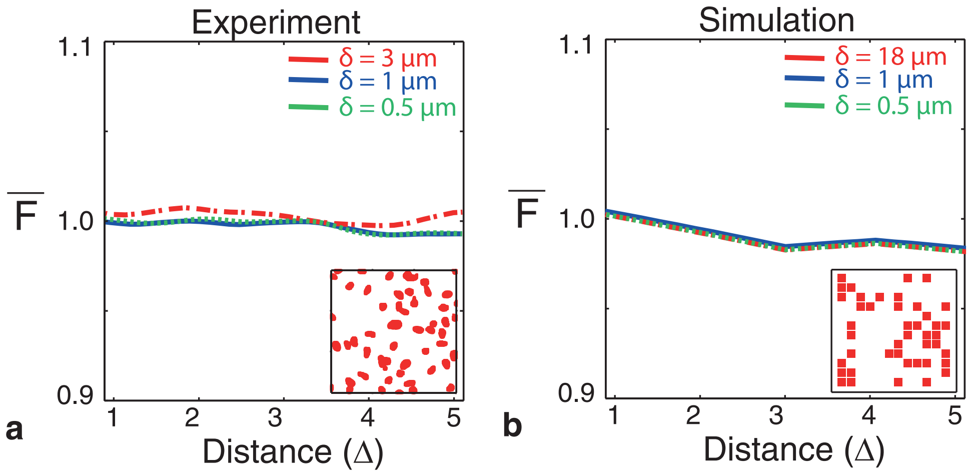

Figure S5. **Size of pair correlation lattice spacing, δ does not affect the pair correlation signal**. Experimental and discrete simulation images were discretised onto a pair correlation lattice with various values of δ. Average pair correlation signals were extracted from four subregions, each of dimension 600 μm $\times$ 600 μm, at the centre of the population at $t = 48$ hours from (a) experiments with Mitomycin–C pretreatment and (b) simulations with cell motility ($D = P_m \Delta^2/4\tau = 248$ μm^2/hour), weak adhesion ($q = 0.3$) and no proliferation. Snapshots of the entire subregion analysed are shown as an inset. The physical size of the inset is approximately 215 μm $\times$ 215 μm. All experiments and simulations were initialised with approximately 30,000 cells or simulated cells, respectively. The green dotted lines correspond to average pair correlation signals computed on a pair correlation lattice with $\delta = 0.5$ μm. Summarily, blue solid lines indicate $\delta = 1$ μm and red dashed lines illustrate results with $\delta = 3$ μm for the experimental images and $\delta = 18$ μm for the discrete simulation images. The horizontal axis is measured as multiples of the average diameter of the nucleus.

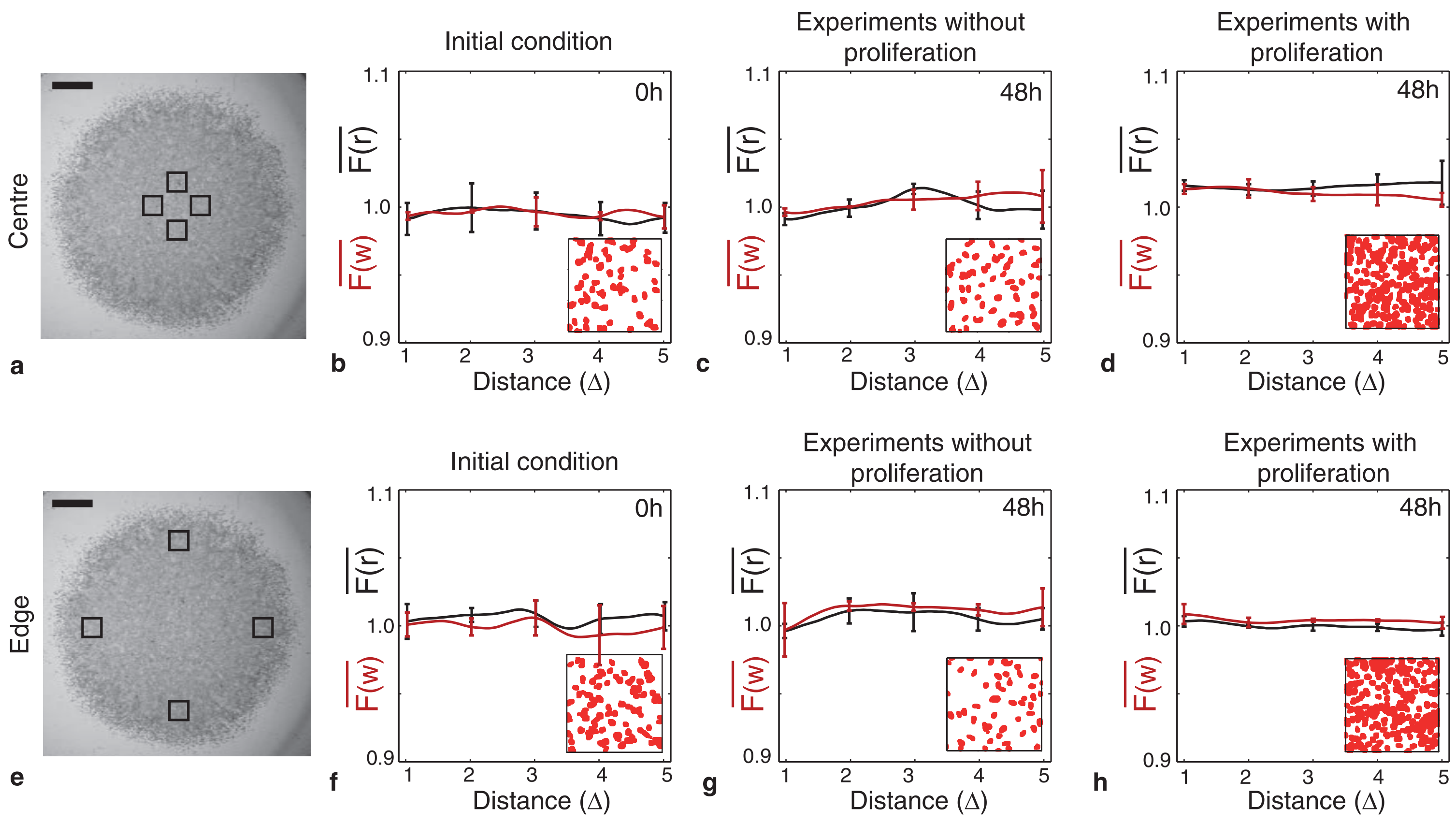

Figure S6. **Comparing the average pair correlation function in the w direction with the average pair correlation function in the r direction**. Average pair correlation functions were extracted from images showing the location of individual cells in four subregions, each of dimension 600 μm $\times$ 600 μm, at the centre of the spreading cell population (a) and four subregions, each of dimension 600 μm $\times$ 600 μm, at the edge of the spreading cell population (e). The relative size and approximate location of these subregions is shown in (a) and (e), respectively, where the scale bar corresponds to 1,500 μm. Pair correlation signals were computed by considering the pair distances of pixels in both the r direction (black, $F(r)$) and in the w direction (red, $F(w)$). Average pair correlation signals are shown at $t = 0$ hours in (b) and (f), at $t = 48$ hours for experiments without cell proliferation in (c) and (g), and at $t = 48$ hours for experiments with cell proliferation in (d) and (h). Results in (b–d) and (f–h) correspond to pair correlation signals computed at the centre and at the edge of the spreading cell population, respectively. The horizontal axis is measured as multiples of the average diameter of the nucleus. Snapshots of the experimental subregions after image processing are shown as an inset. The size of the inset is approximately 215 μm $\times$ 215 μm. Each pair correlation signal was averaged over 12 subregions of dimensions 600 μm $\times$ 600 μm, using three identically prepared experimental replicates. The error bars correspond to one standard deviation about the mean ($N = 12$). All experiments were conducted by initially placing approximately 30,000 cells inside the barrier assay.
